# Improved Activation and Hemodynamic Response Function of Olfactory fMRI Using Simultaneous Multislice with Reduced TR Acquisition

**DOI:** 10.1155/2021/9965756

**Published:** 2021-12-29

**Authors:** Hong Chen, Jianzhong Yin, Che He, Yalin Wu, Miaomiao Long, Guoping Liu, Hongyan Ni, Hua Jin, Yawu Liu

**Affiliations:** ^1^The First Central Clinical College of Tianjin Medical University, Tianjin 300070, China; ^2^Department of Radiology, Tianjin First Central Hospital, Tianjin Medical Imaging Institution, Tianjin 300110, China; ^3^Department of Neurology, Tianjin First Central Hospital, Tianjin 300110, China; ^4^Faculty of Psychology, Tianjin Normal University, Tianjin 300380, China; ^5^Departments of Neurology and Clinical Radiology, Kuopio University Hospital, University of Eastern Finland, Kuopio 33100, Finland

## Abstract

**Objectives:**

The respiration could decrease the time synchronization between odor stimulation and data acquisition, consequently deteriorating the functional activation and hemodynamic response function (HRF) in olfactory functional magnetic resonance imaging (fMRI) with a conventional repetition time (TR). In this study, we aimed to investigate whether simultaneous multislice (SMS) technology with reduced TR could improve the blood oxygen level-dependent (BOLD) activation and optimize HRF modeling in olfactory fMRI.

**Methods:**

Sixteen young healthy subjects with normal olfaction underwent olfactory fMRI on a 3T MRI scanner using a 64 channel head coil. FMRI data were acquired using SMS acceleration at three different TRs: 3000 ms, 1000 ms, and 500 ms. Both metrics of BOLD activation (activated voxels, mean, and maximum *t*-scores) and the HRF modeling (response height and time to peak) were calculated in the bilateral amygdalae, hippocampi, and insulae.

**Results:**

The 500 ms and 1000 ms TRs both significantly improved the number of activated voxels, mean, and maximum *t*-score in the amygdalae and insulae, compared with a 3000 ms TR (all *P* < 0.05). But the increase of these metrics in the hippocampi did not reach a statistical significance (all *P* > 0.05). No significant difference in any BOLD activation metrics between TR 500 ms and 1000 ms was observed in all regions of interest (ROIs) (all *P* > 0.05). The HRF curves showed that higher response height and shorter time to peak in all ROIs were obtained at 500 ms and 1000 ms TRs compared to 3000 ms TR. TR 500 ms had a more significant effect on response height than TR 1000 ms in the amygdalae (*P* = 0.017), and there was no significant difference in time to peak between TR 500 ms and 1000 ms in all ROIs (all *P* > 0.05).

**Conclusions:**

The fast image acquisition technique of SMS with reduced TR could significantly improve the functional activation and HRF curve in olfactory fMRI.

## 1. Introduction

Functional magnetic resonance imaging (fMRI) has been widely used in evaluating brain functions. However, the application of olfactory fMRI is still challenging due to many methodological and physiological factors, such as complex odor stimulation paradigm [1], magnetic susceptibility artifact at the skull base [2], odor adaptation [3], and especially a subject's respiration [4]. All these factors result in a poor blood oxygen level-dependent (BOLD) activation and consequently deteriorate the modeling of hemodynamic response function (HRF) in olfactory fMRI. The respiration gating technology has been employed to decrease the impact of respiration on olfactory fMRI data [5, 6], and the increased activation and a better HRF modeling have been obtained. However, due to respiration triggering, the prolonged odor stimulation paradigm increases the difficulty to accomplish the olfactory task, especially for children and patients with neurodegenerative disorders who are unable to have consistent respiration amplitude. Moreover, respiration gating causes the inconsistent onset timing of odor delivery for different subjects, which may increase the complexity of postprocessing in fMRI data analysis. Therefore, improvement of BOLD activation and optimization of HRF modeling are needed for a better olfactory application.

Olfactory fMRI is conventionally performed using echo planar imaging (EPI) sequences with the standard repetition time (TR) of 2-3 s for whole brain mapping. The fast image acquisition technique of simultaneous multislice (SMS) employs multiband excitation pulses to simultaneously excite and acquire multiple slices [7]. SMS acceleration factor is defined as the number of simultaneously excited slices. The development of SMS technology makes it possible to significantly shorten volume TR, providing the temporal resolution of subsecond [8]. The benefits of reduced TR to improve statistical power through a large amount of data sampling have been confirmed in rest-state fMRI [9]. Moreover, improved BOLD sensitivity by the reduction of TR (<1 s) is found in several task fMRI studies, such as motor fMRI [10]. To our knowledge, only one olfactory fMRI study uses SMS echo planar imaging sequence to investigate the effect of TR on HRF modeling and activated voxels [11], and the result reveals the potential of short TR to improve the activation and the HRF modeling. However, the two tested TRs (901 ms and 1340 ms) in that olfactory fMRI study were within a narrow range and both shorter than the standard TR of a 2D EPI sequence. To fully understand the effect of different TRs on olfactory fMRI, we expanded the tested TR range in the study design.

The primary goal of the study was to investigate whether SMS technology with reduced TR could improve the BOLD activation and optimize HRF modeling in olfactory fMRI.

## 2. Material and Methods

### 2.1. Participants

Sixteen young healthy subjects (mean age ± standard deviation: 25.02 ± 2.5 years; 7 females, 9 males) were recruited into the study. All subjects were right-handed. The inclusion criteria were the age range of 20-30 years and normal olfaction. Olfactory evaluation was performed by T&T olfactory test [12] prior to fMRI examination. Normal olfactory function was defined as the olfactory recognition threshold from -2 to 1 in the T&T olfactory test. All participants had normal olfaction, and their average olfactory recognition threshold score was 0.1 ± 0.4 (mean ± standard deviation). The exclusion criteria were colds or allergies, head trauma, history of nasal surgery, neurological disorders, smoking history, and severe claustrophobia. No subjects were excluded from the study. The study was approved by local institutional review board. All subjects gave written informed consent.

### 2.2. Image Acquisition

All imaging examinations were performed on a 3T MRI scanner (MAGNETOM Prisma, Siemens Healthineers, Erlangen, Germany) using a 64-channel head coil. Structural images were acquired using a T1-weighted magnetization-prepared rapid gradient-echo (MPRAGE) sequence with following imaging parameters: sagittal orientation, TR = 1550 ms, echo time (TE) = 2.98 ms, field of view (FOV) = 256 mm, matrix = 256 × 256, flip angle = 9°, slice thickness = 1.00 mm, number of slices = 176, no gap, effective TI = 900 ms, Generalized Autocalibrating Partially Parallel Acquisition (GRAPPA) acceleration 3, and acquisition time = 2 min 35 s. To investigate the effects of TR on BOLD activation and HRF modeling, three sets of fMRI data were acquired during the olfactory task using three SMS echo planar imaging (SMS-EPI) sequences with different TRs (500 ms, 1000 ms, and 3000 ms). Three corresponding SMS factors were 8, 4, and 1, respectively. Other imaging parameters of the SMS-EPI sequences were kept identical: transverse orientation, TE = 30.0 ms, spatial resolution = 2.4 × 2.4 mm^2^, FOV = 220 mm, matrix = 220 × 220, flip angle = 70°, slice thickness = 2.5 mm, number of slices = 32, and scan time = 11 min 30 s. Based on fMRI scanning time of 11 min 30 s, the total number of frames/scans was set to 1380, 690, and 230 for TRs of 500 ms, 1000 ms, and 3000 ms, respectively. The SMS EPI images were employed with slice GRAPPA image reconstruction.

### 2.3. Olfactory fMRI Paradigm

**Figure 1:**
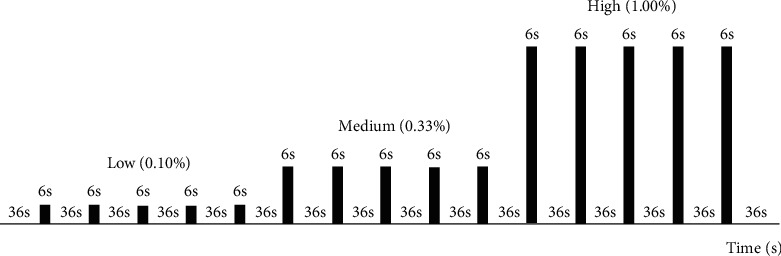
Odor stimulation paradigm. Three different concentrations of lavender: low (0.10%), medium (0.33%), and high (1.00%), are presented. Each concentration is repeated five times before the next higher concentration. Each odor stimulus is 6 s. The interstimulus interval is 36 s with odorless air. Olfactory task is initiated with a 36 s odorless air. The total time of olfactory task is 666 s.

The odor stimulation paradigm was programmed in an MRI-compatible olfactometer (Emerging Tech Trans, LLC, Hershey, PA). The olfactory task was performed with an event-related design, as displayed in Figure 1. Lavender oil (Givaudan Flavors Corporation, East Hanover, NJ) was diluted into odorless distilled water as the odorant. Lavender was an effective odorant with minimal trigeminal stimulation [5]. To compensate the olfactory habitation, three different concentrations of the odor: low (0.10%), medium (0.33%), and high (1.00%) (volume/volume), were successively delivered from low to medium to high concentration [13]. Each odor concentration repeated five times before the next higher concentration. A total of fifteen odor stimulations were presented in olfactory task. Each odor stimulus lasted for 6 s. The interstimulus interval was 36 s with odorless air. The olfactory task was started with a 36 s odorless air. The total olfactory task duration was 666 s. The olfactory task was repeated for each TR condition. Each subject underwent three SMS-EPI scans with the same olfactory task on three adjacent days. And the order of SMS-EPI sequence was randomized for each subject.

The olfactometer delivered constant airflow of 6 L/min at room temperature (22°C). Prior to the fMRI scanning session, all subjects followed the instruction and received the practical training to breathe steadily and regularly through their nose without sniffing. A respiratory belt was attached to the lower sternal angle to closely detect the subject's respiration. Respiration cycle and timing of odor delivery were recorded by the olfactometer.

### 2.4. Data Processing

#### 2.4.1. Preprocessing

All fMRI data preprocessing was performed using Statistical Parametric Mapping (SPM8, http://www.fil.ion.ucl.ac.uk/spm) on Matlab 2013a (Mathworks, USA). The standard data preprocessing procedures were performed identically for three sets of fMRI data: image realignment, coregistration of functional and anatomical images, spatial normalization in the Montreal Neurological Institute (MNI) template space, and smoothing with a Gaussian kernel of 8 mm full width at half maximum (FWHM). A cutoff low-pass filter of 0.01~0.1 Hz was applied to remove high-frequency signal from the fMRI data. No subjects were excluded from data analysis due to the head motion (more than 2 mm of displacement or 1° of rotation in any direction).

#### 2.4.2. Region of Interest (ROI)

**Figure 2:**
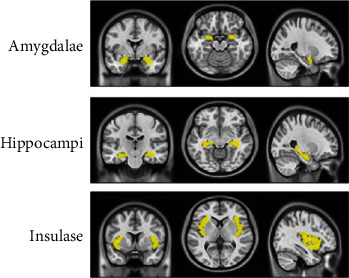
Three regions of interest (ROIs). The templates of the bilateral amygdalae, hippocampi, and insulae are generated based on the anatomical Automatic Anatomical Labelling (AAL) atlas. Three ROIs are displayed in the coronal, transverse, and sagittal planes on Montreal Neurological Institute (MNI) template space.

The amygdala, hippocampus, and insula were three common olfactory-related brain regions [14]. The bilateral amygdalae, hippocampi, and insulae were defined as ROIs. The templates of three ROIs were automatically created using the PickAtlas software and were based on the segmented standard brain atlas from Anatomical Automatic Labeling (AAL) brain [15]. The templates of three ROIs are presented in Figure 2.

#### 2.4.3. General Linear Model (GLM) Analysis

For postprocessing, all smoothed fMRI data were analyzed with general linear model (GLM) and ROI analysis. GLM analysis and ROI analysis were repeatedly performed for each TR condition. In the traditional GLM analysis, a statistical parametric map at the individual level was generated by fitting the olfactory stimulation paradigm to the functional data. The odor stimulations by the three lavender concentrations were used as one stimulation condition [16]. To measure olfactory activation, the random effects model only included the contrast estimation from the odor stimulation against air condition. Voxel-wise *t*-tests were performed to detect activation in olfactory brain regions during the olfactory task (*P* < 0.05, uncorrected, extent threshold = 0). The templates of three ROIs were then applied to the statistical parametric map. The spatial distribution of activation extent (the number of activated voxels) and intensity (the mean and maximum *t*-score) in each ROI were calculated from the statistical parametric map at the individual level. The olfactory activation map at group level was generated using one-sample *t*-tests (*P* < 0.01, uncorrected, extent threshold = 0) across all subjects. The group activation maps were displayed on the high-resolution T1-weighted image using the software of Data processing & Analysis for Brain Imaging (DPABI, https://rfmri.org/dpabi).

#### 2.4.4. ROI Analysis

ROI analysis was performed for smoothed fMRI data to extract the event-related time course in three ROIs using the toolbox of Data processing & Analysis for Brain Imaging (DPABI, https://rfmri.org/dpabi) on Matlab 2013a. The BOLD signal time course represented the percentage changes of the BOLD signal by time [17]. Fifteen BOLD signal time courses after each odor stimulation onset were identified. These BOLD signal time courses were averaged to produce an average BOLD signal time course at each TR for each participant. The averaged BOLD signal time course was used to represent the modeled HRF curve. The following metric of the modeled HRF curve was calculated separately: response height and time to peak. Response height was calculated as the maximum percent signal change [18]. Time to peak was defined as the time for the signal to reach its maximum value [19]. Finally, these values were averaged over all participants.

### 2.5. Statistical Analysis

Statistical analysis was performed using SPSS version 23 (IBM, Armonk, New York, USA). All metrics (activated voxels, mean and maximum *t*-score, response height, and time to peak) were expressed as the mean ± standard deviation. The repeated measures analysis of variance (ANOVA) was performed to examine the effects of TR on each metric. Pairwise comparison testing with Bonferroni's correction was carried for multiple comparisons. The *P* value was defined as smaller than 0.05 to be statistically significant.

## 3. Results

**Figure 3:**
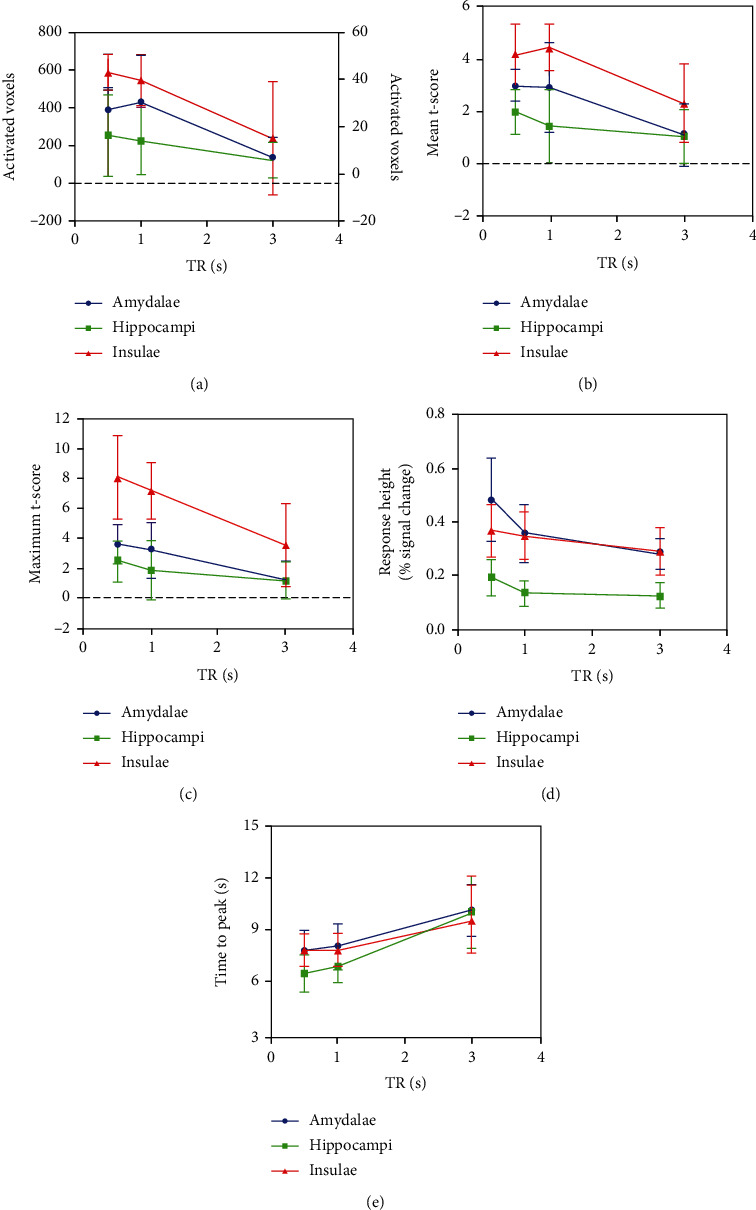
All metrics of different TRs. (a) Activated voxels. (b) Mean *t*-score. (c) Maximum *t*-score. (d) Response height. (e) Time to peak. These metrics averaged across all participants are presented as a function of TR. All error bars indicate standard deviation.

Each subject successfully completed three SMS-EPI scans. All subjects remained awake and breathing calmly during the fMRI scan. Statistical analysis of activated voxels, mean and maximum *t*-score, response height, and time to peak for each ROI are summarized in Table 1. All metrics are plotted as a function of TR in Figure 3 for each ROI.

### 3.1. BOLD Activation of Different TRs

#### 3.1.1. Activation Maps and Activated Voxels

**Figure 4:**
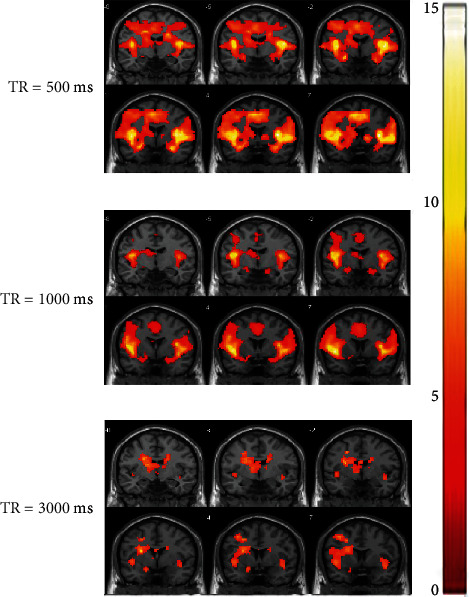
Group activation maps at three different TRs. The group activation maps (*P* < 0.01, uncorrected, extent threshold = 0) are displayed on the T1-weighted anatomical image. The brain activation showed an increasing trend when decreasing TR from 3000 ms to 1000 ms and 500 ms. The color legend represents varying *t*-scores.

Group activation maps of three different TRs are shown in Figure 4, which showed that the activation was significantly larger at TR 500 ms and 1000 ms compared to TR 3000 ms. Repeated measures ANOVA test revealed a significant effect of TR on the activated voxels in the amygdalae (*F*(2, 30) = 18.307, *P* < 0.001) and insulae (*F*(2, 30) = 15.738, *P* = 0.001). As shown in Table 1 and Figure 3(a), in the amygdalae, the number of activated voxels was increased when decreasing TR 3000 ms to 1000 ms (6.81 ± 8.53 vs. 30.06 ± 19.57; *P* = 0.001) and 500 ms (6.81 ± 8.53 vs. 27.06 ± 9.26; *P* < 0.001). In the insulae, the number of activated voxels was increased when decreasing TR 3000 ms to 1000 ms (239.63 ± 297.29 vs. 544.81 ± 133.30; *P* = 0.003) and 500 ms (239.63 ± 297.29 vs. 582.63 ± 96.50; *P* = 0.001). Pairwise comparison testing revealed there was no significant difference in the number of activated voxels between TR 500 ms and 1000 ms in the amygdalae and insulae (all *P* = 1.000). The more activated voxels in the hippocampus were at TR 500 ms and 1000 ms than TR 3000 ms but did not reach a statistic significance (*P* = 0.422).

#### 3.1.2. Mean and Maximum *t*-Score

TR had a statistically significant effect on the mean *t*-score in the amygdalae (*P* < 0.001) and insulae (*P* < 0.001), and no significant effect was observed in the hippocampi (*P* = 0.077), which are shown in Table 1 and Figure 3(b).

The effect of TR on the maximum *t*-score was consistent with it on the mean *t*-score. The maximum *t*-score increased significantly when decreasing TR in the amygdalae and insulae (all *P* < 0.001) but did not significantly change across TRs in the hippocampi (*P* = 0.082), which are shown in Table 1 and Figure 3(c).

Pairwise comparison testing revealed that the mean and maximum *t*-scores were both significantly increased at TR 500 ms and 1000 ms than that at TR 3000 ms in the amygdalae and insulae (all *P* < 0.05). The maximum and mean *t*-scores did not significantly differ between TR 500 ms and 1000 ms in all three ROIs (all *P* > 0.05, Table 1).

### 3.2. HRF Curves of Different TRs

**Figure 5:**
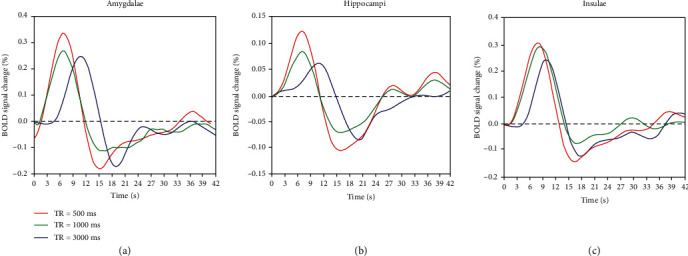
BOLD signal time courses for each TR. The BOLD signal time course represents percentage signal changes of BOLD signal by time. BOLD signal time courses are illustrated separately in the amygdalae, hippocampi, and insulae. Red, green, and blue solid lines are plotted for TRs of 500 ms, 1000 ms, and 3000 ms, respectively.

The HRF curves at different TRs are plotted in each ROI, as shown in Figure 5. Visual analysis of the modeled HRF curve in the amygdalae showed that the BOLD response was indeed presented a steep BOLD signal increase for a short time followed by a decrease with signal values below baseline. The HRF curve in the hippocampi and insulae demonstrated a similar BOLD signal time course.

#### 3.2.1. Response Height

Response height was significantly enhanced when decreasing TR in all ROIs (*P* < 0.001 for the amygdalae, *P* = 0.001 for the hippocampus, and *P* = 0.014 for the insulae) (Table 1 and Figure 3(d)). In the amygdalae, the response height was significantly higher at TR 500 ms than that at TR 3000 ms (*P* = 0.002) and 1000 ms (*P* = 0.017), and the response height did not significantly differ between TR 1000 ms and 3000 ms (*P* = 0.269). In the hippocampi, the response height was only higher at TR of 500 ms compared to TR of 3000 ms (*P* = 0.008) and the other pair comparisons of TRs did not reach statistical differences (all *P* > 0.05). In the insulae, the response height was higher at TRs of 500 ms (*P* = 0.019) and 1000 ms (*P* = 0.035), compared with TR of 3000 ms. There was no significant difference in response height between TR 500 ms and 1000 ms in insulae (*P* = 1.000).

#### 3.2.2. Time to Peak

As presented in Table 1 and Figure 3(e), the time to peak was shortened significantly when decreasing TR in all three ROIs (all *P* ≤ 0.001). In the amygdalae, the averaged time to peak was, respectively, decreased to 8.00s and 7.75 s at TR 1000 ms (*P* = 0.003) and 500 ms (*P* = 0.001) from 10.13 s at TR 3000 ms. Pair comparison testing revealed that the averaged time to peak in the hippocampi was reduced from 10.00s to 6.88 s and 6.44 s when decreasing TR 3000 ms to 1000 ms (*P* = 0.001) and 500 ms (*P* < 0.001), respectively. The averaged time to peak in the insulae was reduced from 9.56 s to 7.81 s and 7.81 s when decreasing TR 3000 ms to 1000 ms (*P* = 0.039) and 500 ms (*P* = 0.013), respectively. The time to peak did not significantly differ between TR 1000 ms and 500 ms in all ROIs (all *P* > 0.05).

## 4. Discussion

The present study investigated whether SMS technology with reduced TR could improve the BOLD activation and optimize HRF modeling in the olfactory fMRI. The results showed that an improved BOLD activation (increased number of activated voxels, mean and maximum *t*-scores) was obtained with reduced TRs (500 ms and 1000 ms), compared with TR of 3000 ms. More than that, higher response height and shorter time to peak in HRF curve were measured with reduced TRs (500 ms and 1000 ms).

In the olfactory fMRI scan, other imaging parameters of three SMS-EPI sequences were equal except for TR. The effect of TR was elucidated. The difference in the number of samples was occurred across three different TR conditions. Shorter TR significantly increased the number of samples in a given time. The fast sampling rate at TR of 500 ms and 1000 ms increased, respectively, the number of images by 6 times and 3 times at the same scan duration, compared with the TR of 3000 ms. The increased amount of data acquisition consequently improved the detection of functional activation. This study result demonstrated that larger activated voxels were measured with TRs of 500 ms and 1000 ms. Georgiopoulos et.al [11] investigated the implication of stimulation length and TR on olfaction activation in olfactory cortex. Their result showed that the combination of short stimulation and short TR (901 ms) could result in more extensive activation. When computing statistical parametric maps at the single-subject level, the degrees-of-freedom differed across three TRs acquisition. Since there was a substantial gain in degrees of freedom at TRs of 500 ms and 1000 ms, the mean and maximum *t*-scores were naturally much higher, showing the increased statistical power. These observations in our olfactory fMRI study were consistent with many other task-based fMRI studies which suggested the benefits of reduction of TR (<1 s) in improving functional activation and statistical power [10, 20, 21].

In this olfactory fMRI, TR 500 ms did not seem to have obvious advantages over TR 1000 ms for improving functional activation. The steady-state MR signal was reduced at shorter TRs. At the short TR acquisition, image signal was exponentially reduced due to decreased T1 recovery within the TR [10, 22]. Therefore, a shorter TR might have a punitive effect on image signal levels.

The sampling density of BOLD response was determined by the TR of the fMRI sequence. SMS technology provided the higher sampling rate at TRs of 500 ms and 1000 ms, compared with 3000 ms. A high data sampling of the BOLD response was advantageous for precisely characterizing the peak signal [11]. The results of this study suggested that the precision of the determination of the HRF peak was better with TRs of 500 ms and 1000 ms than TR 3000 ms. The finding was in accordance with several previous studies on this topic. For example, Dilharreguy et.al [23] evaluated the choice of the TR on the accuracy of the estimation of the peak of the HRF, and the result demonstrated that the choice of the TR influenced the determination of the HRF peak, indicating the position of the HRF peak was calculated with less precision for longer TR. In our study, the result confirmed that TRs of 500 ms and 1000 ms were associated with higher response height in all three olfactory brain regions. This result was consistent with previous results that more pronounced signal increase was thought by giving more densely sampled information of the BOLD response [11, 24]. Faster TRs using multislice EPI sequences could allow more accurate representation of the BOLD response due to more captured information per time unit [24]. For characterizing the differences of the hemodynamic responses between brain regions or between healthy volunteers and patients, reduced TRs of SMS-EPI sequence might be an alternative [23].

The optimum value of TR in the fMRI experiment was not easily calculated. Temporal correlations of the noise limited the very short-TR imaging [25]. Several studies had investigated the optimal TR of SMS sequences for task fMRI, but the difference of optimal TR was presented. The short TR within the range of 300 ms to 600 ms was recommended for more captured information per unit time and more accurate representation of the BOLD response [24]. On the other hand, moderate reduction of TR (1000 ± 200 ms) was also chosen for greater sensitivity and specificity in signal subject event-related fMRI [22]. The difference of optimal TR was thought to be caused by the differences in the experimental design, SMS acquisition parameters, and fMRI data analysis measures.

Bilateral hippocampi were poorly activated for the olfactory stimulation in the present study. The activation in the hippocampi was not drastically increased when decreasing TR. The function and location of the hippocampi might explain something. The hippocampi were classified into the secondary olfactory cortex, involving in higher-order odor-related processing, such as behavior regulation and emotional response [26, 27]. In addition olfaction, hippocampi were involved in episodic memory [28]. Hippocampi were located deeply inside the temporal lobe and were vulnerable to magnetic susceptibility artifact.

The present study had certain limitations. The first limitation was the small size of subjects, and all the subjects were young healthy. Further studies on elder subjects or clinical patients were still needed. Second, we only tested three TRs (500 ms, 1000 ms, and 3000 ms); more TRs within 500 ms-1000 ms should be added into the tested TRs to determine the optimal TR for the BOLD activation and HRF curve. Third, a simple odor stimulation paradigm without respiration triggering was employed in the present olfactory fMRI given that the respiration-triggering method prolonged olfactory stimulation paradigm and increased complexity of data processing. Odor molecules only be sensed when entering into nasal cavity and activating the olfactory epithelium at the beginning of the inspiration phase. The correction of actual odor stimulation onsets as the start time of first inhalation during the odor delivery time period would minimize the time synchronization error between odor delivery and respiration. Reduced TRs using SMS imaging and respiration correction method might further improve BOLD activation and HRF modeling and could be explored in the future work.

## 5. Conclusions

SMS provides an excellent opportunity to investigate neuronal responses to brief stimuli. A better understanding of how to best choose TR in order to optimize experimental design is needed. The acceleration technology of SMS allows to significantly reducing TR. Our study suggested decreasing TRs were beneficial for the improvement of BOLD activation and estimation of HRF in olfactory fMRI.

## Figures and Tables

**Table 1:** The metrics of functional activation and HRF curve in the three olfactory brain regions.

ROIs	TRs			*P*
	500 ms	1000 ms	3000 ms	
Amygdalae				
Activation voxels	27.06 ± 9.26^aa^	30.06 ± 19.57^bb^	6.81 ± 8.53^aa,bb^	<0.001
Mean t-score	3.00 ± 0.62^aa^	2.93 ± 1.72^bb^	1.11 ± 1.18^aa,bb^	<0.001
Maximum t-score	3.60 ± 1.33^aa^	3.21 ± 1.87^bb^	1.19 ± 1.26^aa,bb^	<0.001
Response height	0.47% ± 0.16%^aa,c^	0.34% ± 0.11%^c^	0.26% ± 0.06%^aa^	<0.001
Time to peak	7.75 s ± 1.20 s^aa^	8.00 s ± 1.32 s^bb^	10.13 s ± 1.50 s^aa,bb^	<0.001
Hippocampus				
Activation voxels	16.31 ± 17.08	13.94 ± 14.00	5.81 ± 7.60	0.422
Mean t-score	1.97 ± 0.85	1.45 ± 1.39	1.05 ± 1.02	0.077
Maximum t-score	2.50 ± 1.44	1.86 ± 1.98	1.15 ± 1.22	0.082
Response height	0.17% ± 0.07%^aa^	0.11% ± 0.05%	0.10% ± 0.05%^aa^	0.001
Time to peak	6.44 s ± 1.08 s^aa^	6.88 s ± 0.96 s^bb^	10.00 s ± 2.10 s^aa,bb^	<0.001
Insulae				
Activation voxels	582.63 ± 96.50^aa^	544.81 ± 133.30^bb^	239.63 ± 297.29^aa,bb^	<0.001
Mean t-score	4.20 ± 1.19^aa^	4.45 ± 0.87^bb^	2.32 ± 1.51^aa,bb^	<0.001
Maximum t-score	8.08 ± 2.80^aa^	7.23 ± 1.88^bb^	3.54 ± 2.82^aa,bb^	<0.001
Response height	0.35% ± 0.10%^a^	0.33% ± 0.09%^b^	0.27% ± 0.09%^a,b^	0.014
Time to peak	7.81 s ± 0.95 s^a^	7.81 s ± 0.98 s^b^	9.56 s ± 1.97 s^a,b^	0.001

All metrics are expressed as the mean ± standard deviation. A repeated measures ANOVA is used to estimate the effect of TR. ^a^Statistically significant differences between TRs of 500 ms and 3000 ms; ^a^*P* < 0.05 and^aa^*P* < 0.01. ^b^Statistically significant differences between TRs of 1000 ms and 3000 ms; ^b^*P* < 0.05 and^bb^*P* < 0.01. ^c^Statistically significant differences between TRs of 500 ms and 1000 ms; ^c^*P* < 0.05 and^cc^*P* < 0.01.

## Data Availability

The data that support the findings of this study are available on request from the corresponding author. The data are not publicly available due to privacy restrictions.
